# Salt Reduction Interventions in Sub-Saharan Africa: A Systematic Review

**DOI:** 10.1371/journal.pone.0149680

**Published:** 2016-03-10

**Authors:** Stella Kagwiria Muthuri, Samuel Oji Oti, Richard James Lilford, Oyinlola Oyebode

**Affiliations:** 1 African Population and Health Research Center, P.O. Box 10787-00100, Nairobi, Kenya; 2 Warwick Medical School, University of Warwick, Coventry, CV4 7AL, United Kingdom; University of Perugia, ITALY

## Abstract

**Background:**

Salt intake is associated with hypertension, the leading risk factor for cardiovascular disease. To promote population-level salt reduction, the World Health Organization recommends intervention around three core pillars: Reformulation of processed foods, consumer awareness, and environmental changes to increase availability and affordability of healthy food. This review investigates salt reduction interventions implemented and evaluated in sub-Saharan Africa (SSA).

**Methods:**

MEDLINE and google scholar electronic databases were searched for articles meeting inclusion criteria. Studies that reported evaluation results of a salt intervention in SSA were identified. Titles and abstracts were screened, and articles selected for full-text review. Quality of included articles was assessed, and a narrative synthesis of the findings undertaken. PROSPERO registration number CRD42015019055.

**Results:**

Seven studies representing four countries—South Africa, Nigeria, Ghana, and Tanzania—were included. Two examined product reformulation, one in hypertensive patients and the other in normotensive volunteers. Four examined consumer awareness interventions, including individualised counselling and advisory health sessions delivered to whole villages. One study used an environmental approach by offering discounts on healthy food purchases. All the interventions resulted in at least one significantly improved outcome measure including reduction in systolic blood pressure (BP), 24 hour urinary sodium excretion, or mean arterial BP.

**Conclusions:**

More high quality studies on salt reduction interventions in the region are needed, particularly focused on consumer awareness and education in urban populations given the context of rapid urbanisation; and essentially, targeting product reformulation and environmental change, for greater promise for propagation across a vast, diverse continent.

## Introduction

Cardiovascular disease (CVD) is the leading cause of death globally, with 80% of such deaths occurring in low- and middle-income countries (LMICs) [1]. Evidence shows that high sodium intake may increase blood pressure and consequently lead to hypertension, a leading risk factor for CVD [2, 3]. To promote healthy diets and physical activity, thereby curbing the rise in the prevalence of non-communicable diseases (NCDs), the World Health Assembly adopted the World Health Organisation (WHO) global strategy on diet, physical activity and health in 2004, and later endorsed the global NCD action plan 2008–2013. As part of the implementation of these strategies, WHO’s member states agreed to targets of a 30% and 25% reduction in mean population salt intake and relative reduction in raised blood pressure (systolic ≥140 mmHg and/or diastolic ≥90 mmHg) respectively, in order to meet a 25% reduction in global premature non-communicable disease (NCD) mortality by 2025 [4, 5].

Expert technical meetings were convened to discuss population-level salt reduction strategies, and the result was a recommendation that national programmes employ a multisectoral approach, and be built around three core pillars. The first pillar is *product reformulation* of industrially produced foods. To achieve realistic salt reduction targets, product reformulation ought to be approached in consultation with food producers and distributors, and include monitoring and evaluation, through implementation of mechanisms to track population-level sodium consumption and food sodium composition. Governments were called upon to play a leading role in monitoring and evaluation to this end, including allocation of appropriate resources such as trained staff and an appropriate budget, to facilitate these activities. Several methods were proposed to accomplish effective monitoring including 24 hour or spot urine collections, food diaries, food frequency questionnaires, and the development of food composition databases to allow for accurate assessment of the sodium content in foods. Analysing the sodium content of staple foods, including ethnic foods or foods from restaurants and street vendors, would also be helpful [4–7]. The second pillar is *consumer awareness and education*, which would best be achieved through campaigns targeting individuals or the catering sector, and focusing on knowledge, attitudes, and behaviours regarding salt intake through clear and simple messages. These messaged ought to be tested beforehand, and delivered by identified groups or individuals. It was further recommended that the messages encompass salt that is added during cooking, at the table, or from food consumed outside the home. The avenue of communication used should seek to target not only the general population, but particularly the most vulnerable groups. In addition, provision of information and training on how to read and interpret nutrition labels was also proposed as part of consumer awareness activities [4–6]. The third, *environmental changes*, through setting national targets and standards for food manufacturers and providers, thereby making healthy food choices easy and affordable at the population-level. Clear and comprehensive labelling was also included as a key aspect of environmental change. Experts identified the importance of synergising salt reduction interventions, through policy cohesion and collaboratively working towards policy development, research, monitoring and evaluation, implementation, and advocacy and communication at national, regional, and global levels [4, 5, 8].

Sub-Saharan Africa (SSA)’s populations on average consume more than the recommended sodium intake of 2 g/day [9, 10]. Regrettably, earlier reviews found no national salt reduction initiatives in SSA, with more recent reports finding few existing national-level policies aimed at reducing sodium intake in Nigeria, Mauritius, and South Africa [10–15]. Nigeria’s guidelines on salt intake are broad, advising individuals to limit their intake of salt and bouillon cubes [12]. In Mauritius, a national target to reduce the average sodium intake to 5 g/day, and a strategy centred on food labelling exist as part of the country’s CVD reduction strategy [13]. In 2013, South Africa produced a national strategy to reduce salt intake through mandatory reformulation, which would impact the salt content of processed food and help fight the rising burden of hypertension [14]. While these few and recent developments demonstrate some progress in this area, no national action is being taken towards dietary salt intake reduction in the vast majority of SSA. Additionally, there is no evidence of the effectiveness of the existing national salt reduction policies and interventions.

It is important to note that the types of salt reduction interventions that have been successful in higher income countries may be less effective in LMICs owing to contextual differences [16–18]. It is therefore imperative that before further development and implementation of national policies on salt reduction in SSA, that effectiveness of existing sub-national interventions in this region is examined. Consequently, the objective of this review was to investigate salt reduction interventions conducted in SSA that have been evaluated, and the results published in scientific journals.

## Methods

### Search Strategy, Inclusion and Exclusion Criteria

MEDLINE and google scholar were searched using comprehensive search terms on March 2^nd^ 2015 as shown in Table 1. No date limits were set for the MEDLINE search; however, the google scholar search was restricted to articles published in 1960 onwards. No language or age limits were set for either database. Titles and abstracts of the articles were screened by two independent reviewers, and full text copies obtained for articles meeting the initial screening criteria. Full text articles were then screened in duplicate for inclusion in the review. Studies were included if they reported the results of an evaluation of a salt reduction intervention in a population in SSA. Studies were excluded if the results of an evaluation were not reported, and if the population of interest was not from SSA. Reference lists of included studies were also searched to identify any studies that may have been missed in the initial search. This review is registered with the international prospective register of systematic reviews PROSPERO network, under registration number CRD42015019055.

**Table 1:** Search strategies.

**MEDLINE**	1. Exp Sodium Chloride, Dietary/ or exp Sodium, Dietary/
	2. Salt or sodium
	3. Exp Africa/
	4. 1 OR 2
	5. 3 AND 4
	6. Limit to humans
**Google Scholar**	(Africa Angola Benin Botswana "Burkina Faso" Burundi Cameroon "Cape Verde" "Central African Republic" Chad Comoros Congo "Cote d'Ivoire" Djibouti "Equatorial Guinea" Eritrea Ethiopia Gabon Gambia Ghana Guinea Guinea-Bissau Kenya Lesotho Liberia Madagascar Malawi Mali Mauritania Mauritius Mozambique Namibia Niger Nigeria Reunion Rwanda "Sao Tome and Principe" Senegal Seychelles "Sierra Leone" Somalia "South Africa" Sudan Swaziland Tanzania Togo Uganda "Western Sahara" Zambia Zimbabwe) AND (salt sodium) AND (intervention reduction)

### Data Extraction, Quality Assessment, and Synthesis

Data was extracted from included studies into a spreadsheet under the following headings: author(s), year of publication, year of data collection, country, WHO pillar of intervention(s) (product reformulation, consumer awareness and education, or environmental change), study design, population, intervention, control, outcome measures reported, and results. Data on systolic and diastolic blood pressure, mean arterial pressure (in mmHg) and 24h urinary sodium (in mmol/24h) were extracted, including baseline measurements and results post-intervention, where these were reported. Due to the heterogeneity of study designs, quality was assessed using the quality assessment tool for quantitative studies developed by the Effective Public Health Practice Project [19]. A narrative synthesis of the findings was then undertaken.

## Results

**Fig 1:**
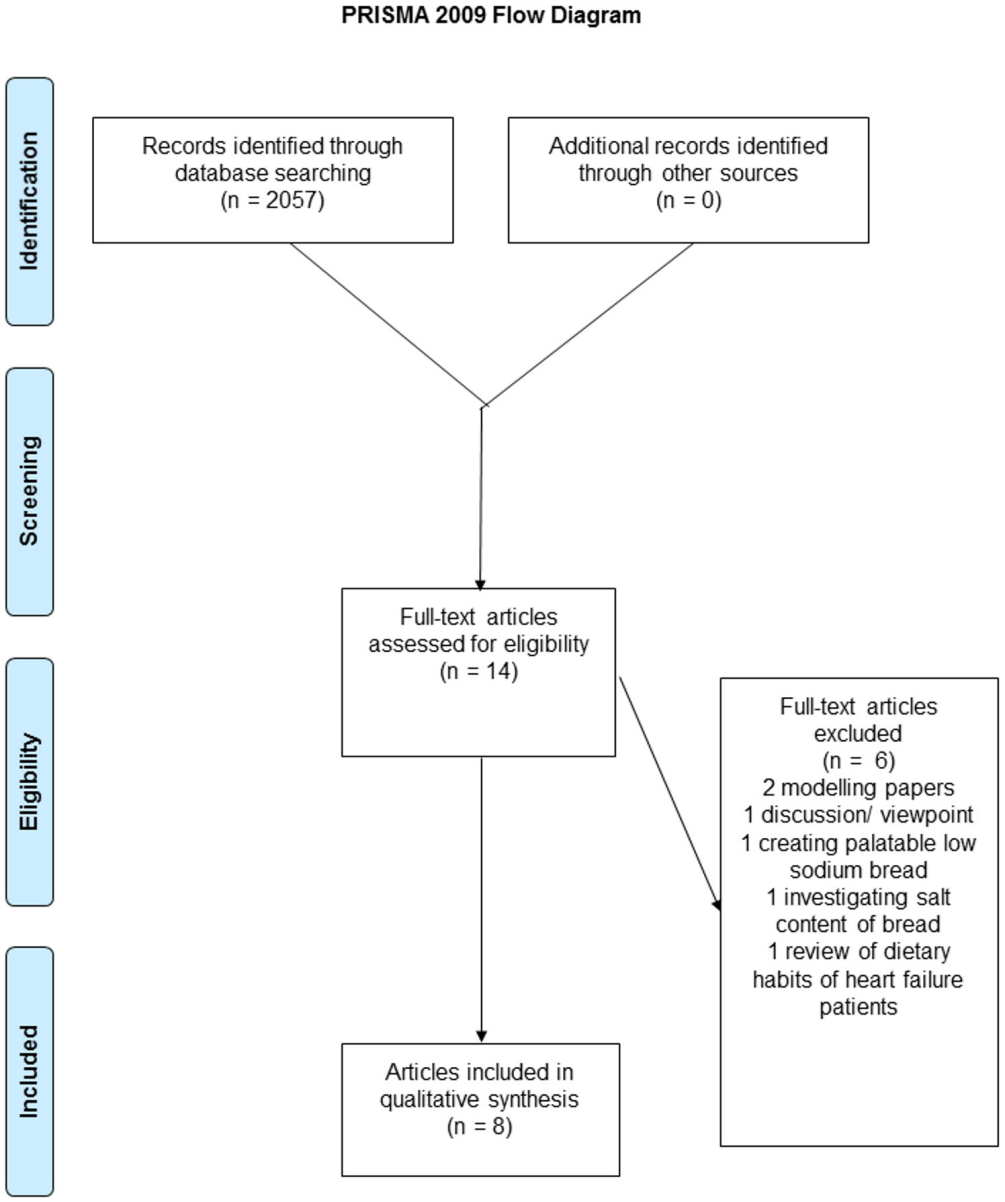
PRISMA Flow Diagram showing inclusion and exclusion of identified papers. *From*: Moher D, Liberati A, Tetzlaff J, Altman DG, The PRISMA Group (2009). *P*referred *R*eporting *I*tems for Systematic Reviews and *M*eta-*A*nalyses: The PRISMA Statement. PLos Med 6(6): e1000097. doi: 10.1371/journal.pmed1000097 For more information, visit www.prisma-statement.org.

Fig 1 shows the PRISMA flow diagram with number of included and excluded studies. A total of 2057 records were identified through the MEDLINE and google scholar databases. Following de-duplication and an initial title and abstract screening process, 14 articles were retrieved for full-text review. Of the 14 articles, eight papers reporting on seven individual studies, were included in this systematic review.

As shown in Table 2, four countries, including South Africa, Nigeria, Ghana, and Tanzania, were represented in these seven studies. The earliest paper was published in 1990 and the most recent published in 2013. The studies include a range of study designs including one cluster randomised controlled trial (RCT), with villages used as clusters; two RTCs; one random cross-over trial (participants on three weeks of low/high salt, two weeks wash-out, and then three weeks on low/high salt); one observational study; and, two studies with no contemporaneous control group (before and after studies). Three studies were of weak quality, and the remainder were moderate. There were no studies designated as high quality in this review.

**Table 2:** Included articles.

Reference	Country	Data Collection Period	WHO Pillar	Study Design	Quality Score
Charlton et al., 2008 [20]	South Africa	May 2004–July 2005	Product Reformulation	RCT	Moderate
Mtabaji et al., 1990 [21]	Tanzania	Not reported	Product Reformulation	RCT	Weak
Adeyemo et al., 2002 [22]	Nigeria	Not reported	Consumer Awareness	Cohort (before-after)	Moderate
Cappuccio et al., 2000 [23]	Ghana	1999	Consumer Awareness	Cohort (before-after)	Moderate
Cappuccio et al., 2006 [24]	Ghana	June 2001–June 2002	Consumer Awareness	Cluster RCT	Weak
Forrester et al., 2005 [25]	Nigeria	Not reported	Consumer Awareness	Randomised crossover trial	Weak
An et al., 2013 [26]; Sturm et al., 2013 [27]	South Africa	Feb 2009–Nov 2011	Environmental Change	Observational	Moderate

**Acronyms**: Randomised Control Trial (RCT)

As shown in Table 3, two studies examined product reformulation [20, 21]. In both studies, the reformulated products were provided to the participants. The RCT by Charlton and colleagues (2008) in hypertensive patients found a significant reduction in systolic blood pressure by 6.2 mmHg, as shown in Table 4, but no corresponding change in diastolic blood pressure or 24 hour urinary sodium excretion. It is noteworthy that in this study, besides sodium concentration reformulation, potassium, magnesium and calcium concentrations were also reformulated (increased) in the products [20]. In the RCT by Mtabaji and colleagues (1990), conducted in normotensive volunteers, a reduction in 24 hour urinary sodium excretion and mean arterial blood pressure (of 6mmHg, p<0.001) was found after 4 to 5 days on the low sodium diet; however, this study was assessed as weak in quality, since a study population of volunteers is not likely to represent a general population and the control arm were given supplementary sodium rather than being on a normal diet. There was also no indication that the study was blinded, and no description on how urinary analysis was determined to be valid or reliable [21].

**Table 3:** Details of included studies.

Reference	Population	Intervention	Control	Outcomes Measured	Results
**Product Reformulation**					
Charlton et al., 2008 [20]	80 Black residents of a Cape Town township aged 50–75 years, with drug-treated mild-to-moderate hypertension but without type 1 diabetes, impaired cognitive function, incontinence, renal impairment, cerebral infarction or haemorrhage, not taking furosemide for cardiac failure, not drinking three or more alcoholic drinks per day, and a BMI under 45.	Sodium, potassium, magnesium and calcium content were modified in 5 commonly consumed food items (brown bread, margarine, stock cubes, aromat (flavour enhancer), and soup mixes. These items plus a salt replacement (solo), fermented milk (maas), and enough food was given to the whole family for 8 weeks (delivered 3 times a week). The subjects were instructed to consume their usual amounts of food.	The same items were provided to a control group of participants, but using the standard commercial compositions, and an artificially sweetened cold drink was given instead of maas.	24h urinary sodium. Systolic and diastolic blood pressure.	No significant reduction in 24h urinary sodium. Reduction in systolic blood pressure. No significant reduction in diastolic blood pressure.
Mtabaji et al., 1990 [21]	30 male normotensive volunteers.	Participants were given a low sodium diet of about 50 mmol/day.	The control arm of participants were given a normal diet supplemented by 250 mmol of sodium in the form of a soup.	24h urinary sodium. Mean arterial pressure.	Reduction in 24h urinary sodium. Reduction in mean arterial pressure.
**Consumer Awareness and Education**					
Adeyemo et al., 2002 [22]	88 participants were randomly selected from a population-based register of adults aged ≥25 years among those who had systolic blood pressure within the top 20% of the population's distribution. Participants were recruited from two rural communities in Idere and Igbo-Ora, and were mainly farmers.	Participants were taken through a counselling and food tasting session teaching them (1) how to reduce salt added during cooking by half, and (2) how to eliminate the use of bouillon cubes and monosodium glutamate seasoning for a 2-week period	Not applicable	24h urinary sodium. Systolic and diastolic blood pressure.	Reduction of 24h urinary sodium. Reduction in systolic blood pressure. No significant reduction in diastolic blood pressure.
Cappuccio et al., 2000 [23]	20 farmers (8 men, 12 women) were selected randomly from households in the village of Odoyefe in the Ashanti region of Ghana.	A week of daily nutrition education (1.5 hours each) was offered, followed by once a week sessions thereafter for 4 weeks.	Not applicable	24h urinary sodium. Systolic and diastolic blood pressure.	Reduction in 24h urinary sodium. Reduction in systolic blood pressure. Reduction in diastolic blood pressure.
Cappuccio et al., 2006 [24]	12 communities (villages) in the Ashanti region, 6 rural and 6 semi-urban.	Community Health Workers delivered sessions using flip charts as the main means of communication. These were held daily for one week and once a week thereafter, each lasting one hour (for both intervention and control arms). The standard health education package included prevention of malaria, infective diarrhoea, roundworm infection, and awareness of diabetes and hypertension. In addition to the standard health education package, additional advice was given to the intervention arm to limit the consumption of 5 salty foods, and when eaten, to soak the items in water overnight beforehand, and not to add salt to food.	Control villages received the standard health education package.	24h urinary sodium. Systolic and diastolic blood pressure. The intervention was carried out with the whole village. The measurements were only taken in a random sample of 1013 participants from these villages.	No significant reduction in 24h urinary sodium. No significant change in systolic blood pressure. Reduction in diastolic blood pressure.
Forrester et al., 2005 [25]	58 participants recruited from the Igbo-Ora and Idere rural communities in South West Nigeria (about 50 miles from Ibadan). They were normotensive men and women aged 25–55 years, who were able to give informed consent, excluding pregnant and breastfeeding women, people with history of diabetes, kidney disease, atherosclerotic vascular disease or obesity (BMI over 40).	For the low-salt diet arm, case managers provided information and counselling to participants to help identify dietary sodium sources and enhance behavioural skills for reducing salt intake. For the high-salt diet arm, participants were instructed to consume their regular diet and take four capsules containing 16 mEq each. Compliance was monitored by a daily log and pill counts at each visit.	A randomisation scheme was constructed in blocks of four. Individuals were allocated to either a low-salt or high-salt diet for 3 weeks, followed by 2 weeks wash-out, then a crossover for an additional 3 weeks.	24h urinary sodium. Systolic and diastolic blood pressure.	Reduction in 24h urinary sodium. Reduction in systolic blood pressure. Reduction in diastolic blood pressure.
**Environmental Change**					
An et al., 2013 [26]; Sturm et al., 2013 [27]	351,319 members of a health and life insurance company’s health promotion programme in 169,485 households.	Everyone enrolled in the health promotion programme was eligible for the Healthy Food Benefit, but to receive it, they were required to activate it online or via a phone call. Those who activated the benefit received 10% off healthy food purchases in a specific supermarket chain (“Pick n Pay”) and 25% off if they completed an online health risk assessment questionnaire.	For analysis of survey data, participants were classified into three groups dependent on the level of benefit at the time of survey completion: 0%, 10% and 25%. For analysis of credit card purchases, participants were classified according to their level of benefit at the time of purchase (therefore some households acted as their own control).	Survey data: “How often do you eat a) high sugar food; b) fried food; c) processed meats; d) fast food” “How salty do you like your food? Not salted, slightly salted, or very salty” Food purchases made in “Pick n Pay” with a Visa credit card.	**Survey results**: The effect on dietary behaviour of the discount on healthy foods is in the expected direction for each food type examined. Individuals receiving a discount were less likely to eat food high in salt, fried food, processed meats or fast food. **Purchase results**: Among those accessing the discount, the ratio of healthy food to total food purchases increased, and the ratio of unhealthy food to total food purchases decreased.

**Acronyms**: Body Mass Index (BMI), 24 hour (24h).

**Table 4:** Quantitative results from included studies.

	Systolic Blood Pressure (mmHg)				Diastolic Blood Pressure (mmHg)				24h urinary sodium (mmol/day)			
	Baseline (μ (SD))		Results (μ (95% CI))		Baseline (μ (SD))		Results (μ (95% CI))		Baseline (μ (SD))		Results (μ (95% CI))	
Charlton et al., 2008 [20]	Intervention	133.9 (14.6)	Control—Intervention	6.2 (1.0–11.4)	Intervention	79.8 (8.6)	Control- Intervention	0.6 (-1.8–3.0)	Intervention	171.7 (53.7)	Post-pre intervention (μ (sd))	-14.6 (54.4) p>0.05
	Control	135.4 (16.7)			Control	82.3 (7.5)			Control	173.2 (52.4)	Control—Intervention (μ (sd))	8.7 (46.9) p>0.05
Mtabaji et al., 1990 [21]											High salt (μ (sd))	324 (25.5)
											Low salt (μ (sd))	52.4 (6.9)
Adeyemo et al., 2002 [22]	Men	116.8 (15.3)	Post-pre intervention	4.7 (1.9–7.4)	Men	74.0 (9.5)	Post-pre intervention	1.9 (-0.3–4.1)	Men	140.5 (53.4)	Post-pre intervention	76.9 (59.7–94.1)
	Women	110.1 (14.6)	Post-pre intervention	7.0 (2.6–11.4)	Women	69.1 (11.5)	Post-pre intervention	1.6 (-1.8–5.0)	Women	132.6 (48.0)	Post-pre intervention	79.4 (59.4–99.5)
Cappuccio et al., 2000 [23]	Total Sample	135.3 (16.5)	Post-pre intervention	6.4 (0.5–12.3)	Total Sample	85.8 (8.6)	Post-pre intervention	4.5 (-0.3–9.3)	Total Sample	99.4 (49.4)	Post-pre intervention	44.1 (22.3–65.9)
Cappuccio et al., 2006 [24]	Intervention	129 (25)	Control—Intervention at 6 months	2.5 (-1.5–6.5)	Intervention	77 (13)	Intervention—Control at 6 months	4.0 (0.8–7.1)	Intervention	99.9 (44.7)	Intervention—Control at 6 months	-6.0 (-16.1–4.1)
	Control	127 (27)			Control	76 (13)			Control	102.5 (45.3)		
Forrester et al., 2005 [25]	Total Sample	114.8 (11.4)	High salt—low salt phase	4.5 (1.6–7.3)	Total Sample	73.3 (9.1)	High salt—low salt phase	2.7 (0.9–4.5)	Pre-intervention/High salt diet	93.0/127.3	High—Low salt diet	93.7

Note: 1mmol sodium = 58.5 mg salt. Mean arterial pressure results are presented in the text.

Four studies examined consumer awareness interventions in rural or semi-rural settings [22–25] [Table 3]. Two studies were resource intensive, including one before and after cohort study by Cappuccio and colleagues (2000), which examined the effectiveness of daily and weekly lessons with participants, and the second, also a before and after cohort study, by Adeyemo and colleagues (2002), which involved one-on-one counselling to help identify participants’ main sources of dietary sodium and modifying behaviour for reductions in salt intake. Both interventions reported a reduction in the mean 24 hour urinary sodium excretion and systolic blood pressure of 6.4mmHg [22] and 4.7mmHg in men and 7.0 mmHg in women [23] [Table 4]). Two studies were less resource intensive; the first, a cluster RCT involving hour-long health advice sessions on salt reduction delivered by community health workers to whole villages daily for one week, and then once a week thereafter, in addition to standard health messaging [24]. The second, a randomised cross-over trial, involving a counselling and food tasting session to teach participants how to reduce salt added during cooking by half and how to eliminate the use of bouillon cubes and monosodium glutamate seasoning for a 2-week period [25]. The first study found a reduction in diastolic blood pressure (4.0mmHg) following the intervention, while the second found a reduction in systolic (4.5mmHg) and diastolic (2.7mmHg) blood pressure as well as urinary sodium [Table 4], although both were assessed as weak in quality the former due to vulnerability to selection bias, lack of intervention integrity (possible contamination) and blinding and the latter also due to selection bias and failure to blind participants and researchers.

Finally, an observational study, reported in two papers, examined environmental change [26, 27] [Table 3]. Members of a life and health insurance scheme who had signed up to receive this benefit received a 10% discount on healthy food purchases, or a 25% discount if they completed a health risk assessment questionnaire online. Discount recipients were then compared to regular members with the finding that discount holders spent more on healthy foods as a proportion of total food expenditure, and less on unhealthy food. They also reported eating healthier foods more often, and unhealthy foods (including salty food, processed meat, fast-food and fried food) less often. These papers did not report any individual health outcomes such as blood pressure measurements or urinary sodium.

## Discussion

The objective of this review was to investigate salt reduction interventions conducted in SSA that have been evaluated, with results published. A search of the MEDLINE and google scholar databases yielded eight articles, representing seven studies, for inclusion in the analyses. Considered the gold-standard for inclusion in systematic reviews, 3 of the studies were RCTs; however, given the limited evidence available, observational studies and other research designs were also included. Due to the small number of studies identified and their diverse nature with respect to study design, intervention, population and outcomes reported, it was not possible to undertake a meta-analyses, which limits the strength of the conclusions that can be drawn.

The product reformulation interventions resulted in reduction of systolic blood pressure [20], 24h urinary sodium excretion [21], and mean arterial pressure [21]. It is important to note that the food items selected for reformulation were commonly consumed foods, delivered to the end user. For an effective population-wide intervention, product reformation of a select number of products that are commonly used by virtually all members of a nation, may be the best approach. States would have to approach this in consultation with key players in their respective food industries, and channel resources towards monitoring product changes and consumer salt intake, as well as evaluation of successes. Customer awareness and education interventions resulted in a reduction in 24 hour urinary sodium excretion [22, 23, 25], systolic blood pressure [22, 23, 25], and diastolic pressure [23, 24, 25]. Individual and group educational sessions and counselling on salt reduction techniques during cooking and limiting salty food intake outside the home, were effective in improving some key outcomes. One important feature of all the included studies was that while the education or counselling sessions were done intensively at first, they all required an extent of follow-up to reinforce the learnings, typically lasting 2 weeks or more. It is also interesting to note that all the consumer awareness and education interventions captured in this review were conducted in rural populations. Rapid urbanisation in SSA has resulted in a vast change in the demographic landscape of the continent with a projected population increase of 0.9 billion by 2050 [28, 29]. Changing dietary habits, proliferation of high-salt content fast foods, and use of preservatives may lead to a growing number of urban dwellers consuming more salt than their rural counterparts [16, 17]. It is therefore imperative that such salt reduction interventions be tested in urban areas. The environmental change intervention, achieved through subsidies on healthy food purchases, led to higher spending on and eating of healthy foods, and consequently lower spending and eating unhealthy and high salt content foods [26, 27].

All the interventions had at least one significantly improved outcome measure; however, it is important to consider the possibility of publication bias, resulting from interventions with no significant health benefits being unreported. Taken together, while 24 hour urinary sodium excretion was reduced in half of the included studies, blood pressure, and most frequently systolic blood pressure, was reduced in all studies in which it was reported. This may suggest that assessment of 24 hour urinary sodium excretion is associated with greater measurement error. Alternatively, it could mean that blood pressure, even in normotensive individuals, may be more sensitive to changes in salt intake. Systolic hypertension is the most common form of hypertension especially in older age groups, systolic blood pressure is almost always less well controlled than diastolic blood pressure, and it has been argued that reduction in systolic blood pressure is more clinically relevant, particularly among the highest risk and older hypertensive patients [30–32].

## Conclusions

Since 2007, the WHO has supported development of national salt reduction strategies by establishing networks and partnerships with regional organisations around the world [33–35]; however, few African countries have national strategies in place to address salt intake [10–15]. Evaluation of high quality studies on salt reduction interventions in the region is also lacking. To attain effective population-wide salt reduction interventions and have larger impact, it is important that policy development target supply chain and main sources of dietary sodium, rather than individual level behaviour change [36]. The former are also less resource intensive per person, and have greater reach. We found only two studies in this category [20, 21], one of which was designated as weak in quality. The majority of studies focused on customer awareness and education, with less promise for scale-up across a vast and diverse continent. One study used financial incentives rather than education to achieve individual-level change and may potentially have broader reach than consumer education. Indeed, comparative cost-effectiveness assessments of these interventions would identify the most viable and affordable option for states in the region. Given the ongoing rapid urbanisation in SSA, it is crucial that such interventions and evaluations be conducted among urban residents, to curb growth in the prevalence of risk factors for, and CVDs.

## Supporting Information

S1 PRISMA Checklist PRISMA Checklist. (DOC)
